# Sick Leave and Factors Influencing Sick Leave in Adult Patients with Atopic Dermatitis: A Cross-Sectional Study

**DOI:** 10.3390/jcm4040535

**Published:** 2015-03-27

**Authors:** Harmieke van Os-Medendorp, Simone Appelman-Noordermeer, Carla Bruijnzeel-Koomen, Marjolein de Bruin-Weller

**Affiliations:** 1 Department of Dermatology/Allergology, University Medical Centre Utrecht, Heidelberglaan 100, 3584CX Utrecht, The Netherlands; E-Mails: c.bruijnzeel@umcutrecht.nl (C.B.-K.); m.s.debruin-weller@umcutrecht.nl (M.B.-W.); 2 Student Clinical Health Sciences, University Medical Centre Utrecht, Heidelberglaan 100, 3584CX Utrecht, The Netherlands; E-Mail: sm_noor@hotmail.com

**Keywords:** atopic dermatitis, sick leave, influencing factors, quality of life, socio-occupational factors, severity

## Abstract

Background: Little is known about the prevalence of sick leave due to atopic dermatitis (AD). The current literature on factors influencing sick leave is mostly derived from other chronic inflammatory diseases. This study aimed to determine the prevalence of sick leave due to AD and to identify influencing factors. Methods: A cross-sectional study was carried out in adult patients with AD. Outcome measures: sick leave during the two-week and one-year periods, socio-demographic characteristics, disease severity, quality of life and socio-occupational factors. Logistic regression analyses were used to determine influencing factors on sick leave over the two-week period. Results: In total, 253 patients were included; 12% of the patients had to take sick leave in the last two weeks due to AD and 42% in the past year. A higher level of symptom interference (OR 1.26; 95% CI 1.13–1.40) or perfectionism/diligence (OR 0.90; 95% CI 0.83–0.96) may respectively increase or decrease the number of sick leave days. Conclusion: Sick leave in patients with AD is a common problem and symptom interference and perfectionism/diligence appeared to influence it. Novel approaches are needed to deal with symptoms at work or school to reduce the amount of sick leave due to AD.

## 1. Introduction

Atopic dermatitis (AD) is a chronic skin disease with exacerbations and remissions which has a high impact on quality of life [1]. The overall prevalence of AD in general population is 2.3%, with a higher prevalence of 11.3% under children up to six years [2].

Very few studies have analysed the impact of AD on the number of missed work days, and those that have show varying results. In a study in Denmark, the loss of work days due to AD was 5.8 days in a six-month period [3]. An international study showed that 2.7 days were lost per year due to work absence and 9.6% of work time was affected during flares [4]. In a Spanish study, the number of lost work days was 8.3 (sd 23.7) in one year [5]. Sick leave is not only inconvenient for the patient, it also incurs indirect economic costs [4,6,7]. These avoidable indirect economic costs for patients with AD were estimated at €2 billion per year across the EU [4].

To date, it is unclear which factors influence sick leave in patients with AD or other chronic diseases, and reports vary in their findings. The increasing effect of disease severity on sick leave was demonstrated in a cross-sectional study conducted in eight different countries in 2002 patients with AD [4] and in a study on 1673 patients with asthma in Spain [8]. In contrast, a study in the Netherlands by Boot *et al*. [9] reported that severity of disease was not a determinant of sick leave in asthma or COPD patients. For patients with psoriasis, it was also revealed that disease severity was not a predictor of productivity loss [10]. Other factors influencing sick leave in patients with chronic diseases are a low health-related quality of life [9,10], poor disease control [11,12] adaptation and psychosocial variables [13] and visits to health care providers [8].

The aim of this study is to demonstrate the extent of sick leave in the Netherlands due to AD and to explore factors that influence sick leave for these patients.

## 2. Results

### Patient Characteristics

**Figure 1:**
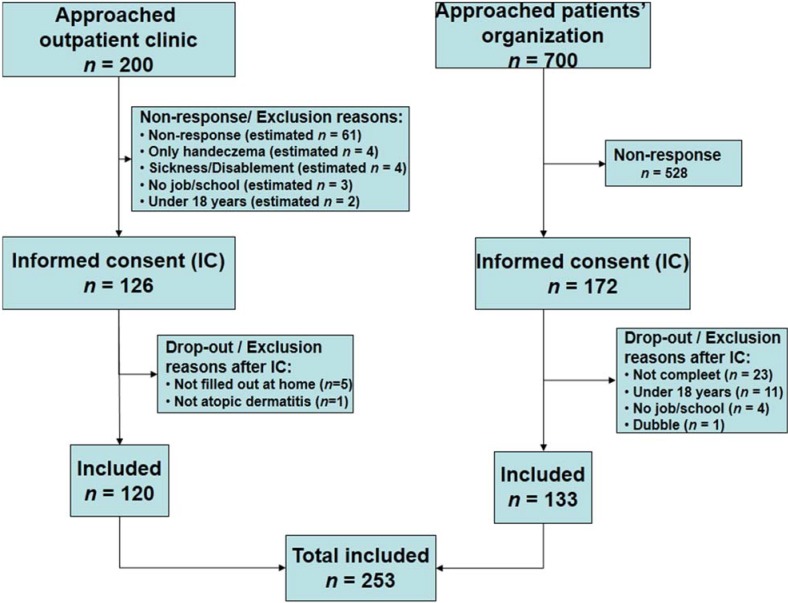
Flowchart inclusion process.

In total, 253 patients were included (Figure 1). The mean age of the study participants was 36.9 (sd 12.4) years; 66% were female and most (49%) had a high educational level. Other chronic diseases were present in 54% of all patients, of which allergies (44%) and pulmonary diseases (36%) were the most common. No differences were observed between the patients from the outpatient department and the patients’ organization except for sex and educational level (Table 1).

## 3. Severity of AD, Quality of Life and Socio—Occupational Complaints

The mean severity score of AD was 15.1 (sd 7.2); most patients had moderate (37%) or severe (35%) to very severe (10%) AD. Of the study population, 44% had a severely impaired quality of life and 76% scored high on the symptoms subscale. Of the social-occupational subscores, 25%–42% patients displayed above mean or high scores for distress, work pressure, job dissatisfaction, little flexibility, stressful home situation, avoidance/uncertainty or perfectionism/diligence; 5% presented above mean or high scores on symptom interference (Table 2). Patients from the patients’ organization scored higher on functioning, a subscale of quality of life.

**Table 1:** Patient characteristics.

Demographics		Total Group- *n* = 253	Outpatient Department- *n* = 120	Patients’ Organization- *n* = 133	Difference Chi Square-*p*/*t*-test-*p*
Sex (*n*, %)	Man	87 (34)	51 (43)	36 (27)	0.010
	Woman	166 (66)	69 (58)	97 (73)	
Age (mean, sd)		36.9 (12.4) (*n* = 252) range 18–70	36.3 (13.3) range 18–70	37.3 (11.4) (*n* = 132) range 18–65	0.52
Disease duration (mean in years, sd)		28.1 (14.0)	27.5 (14.0)	28.7 (14.1)	0.49
Education (*n*, %)	low	29 (12)	21 (18)	8 (6)	0.000
	medium	99 (39)	54 (45)	45 (34)	
	high	125 (49)	45 (38)	80 (60)	
Work or school (*n*, %)	school	15 (6)	9 (8)	6 (5)	0.52
	work	188 (74)	86 (72)	102 (77)	
	combination of work and school	50 (20)	25 (21)	25 (19)	
Other chronic diseases (*n*, %)	Yes	137 (54)	68 (57)	69 (52)	0.45
	No	116 (46)	52 (43)	64 (48)	
Other allergic diseases (hay fever, food allergy) (*n*, %)	Yes	112 (44)	55 (46)	57 (43)	0.63
	No	141 (56)	65 (54)	76 (57)	
Pulmonary diseases	Yes	90 (36)	45 (38)	45 (34)	0.54
	No	163 (64)	75 (63)	88 (66)	

**Table 2:** Severity of AD, quality of life and socio-occupational complaints.

Variable		Total Group (*n* = 253)	Outpatient Department (*n* = 120)	Patients’ Organization (*n* = 133)	Difference Chi square-*p*/*t*-test-*p*
**Severity of Atopic Dermatitis**					
Severity (mean, sd) (POEM)		15.1 (7.2)	14.7 (7.2)	15.5 (7.2)	0.37
Severity (*n*, %)	Clear/almost clear (score 0–2)	8 (3.2%)	5 (4.2%)	3 (2.3%)	
	Mild (score 3–7)	36 (14.2%)	17 (14.2%)	19 (14.3%)	
	Moderate (score 8–16)	94 (37.2%)	47 (39.2%)	47 (35.3%)	
	Severe (score 17–24)	89 (35.2%)	41 (34.2%)	48 (36.1%)	
	Very severe (score 25–28)	26 (10.3%)	10 (8.3%)	16 (12.0%)	
**Quality of Life (Skindex 29)**					
Symptoms (*n*, %)	score < 52	61 (24)	34 (28)	27 (20)	0.14
	score ≥ 52	192 (76)	86 (72)	106 (80)	
Emotions (*n*, %)	score < 39	138 (55)	62 (52)	76 (57)	0.38
	score ≥39	115 (46)	58 (48)	57 (43)	
Functioning (*n*, %)	score < 37	150 (59)	79 (66)	71 (53)	0.04
	Score ≥ 37	103 (41)	41(34)	62 (47)	
Overall score (*n*, %)	score < 44	143 (57)	70 (58)	73 (55)	0.58
	Score ≥ 44	110 (44)	50 (42)	60 (45)	
**Social Occupational Complaints**					
Symptom interference (*n*, %)	low to mean score	241 (95)	116 (97)	125 (94)	0.32
	above mean or high score (≥31)	12 (5)	4 (3)	8 (6)	
Distress (*n*, %)	low to mean score	190 (75)	93 (78)	97 (73)	0.40
	above mean or high score (≥29)	63 (25)	27 (23)	36 (27)	
Work pressure (*n*, %)	low to mean score	147 (58)	73 (61)	74 (56)	0.40
	above mean or high score (≥16)	106 (42)	47 (39)	59 (44)	
Job dissatisfaction (*n*, %)	low to mean score	150 (59)	76 (63)	74 (56)	0.21
	above mean or high score (≥26)	103 (41)	44 (37)	59 (44)	
Little flexibility (*n*, %)	low to mean score	159 (63)	70 (58)	89 (67)	0.16
	above mean or high score (≥17)	94 (37)	50 (42)	44 (33)	
Stressful home situation (*n*, %)	low to mean score	182 (72)	88 (73)	94 (71)	0.64
	above mean or high score (≥15)	71 (28)	32 (27)	39 (29)	
Avoidance/uncertainty (*n*, %)	low to mean score	172 (68)	85 (71)	87 (65)	0.36
	above mean or high score (≥24)	81 (32)	35 (29)	46 (35)	
Perfectionism/diligence (*n*, %)	low to mean score	181 (72)	85 (71)	96 (72)	0.81
	above mean or high score (≥37)	72 (29)	35 (29)	37 (28)	

### 3.1. Prevalence of Sick Leave

In total, 23% of patients took sick leave in the previous two weeks and 64% in the last year. Sick leave due to AD was 12% in the last two weeks and 42% in the last year. The mean number of days of sick leave due to AD in the previous two weeks was 0.3 (sd 0.9) and 5.7 (sd 12.2) days in the last year (Table 3).

### 3.2. Factors Influencing Sick Leave

Univariate logistic regression analysis demonstrated that disease severity, quality of life and socio-occupational factors (symptom interference, distress, job dissatisfaction, stressful home situation, avoidance/uncertainty and perfectionism/diligence) were related to sick leave in the last two weeks (*p* < 0.2) (Table 4). These factors were included in the final, multivariate regression. The full model was statistically significant (chi^2^ 52.5, 8 df, p. 0.00) and explained between 19 (Cox and Snell R square) and 37% (Nagelkerke R square). The final model determined that a higher level of symptom interference, Odds Ratio (OR) 1.26 (95% CI: 1.13–1.40) could increase the number of sick leave days, and higher scores on perfectionism/diligence could decrease the number of sick leave days, OR 0.90 (95% CI: 0.83–0.96) (Table 5).

**Table 3:** Prevalence of sick leave.

Sick Leave		Total Group (*n* = 253)	Outpatient Department (*n* = 120)	Patients’ Organization (*n* = 133)	Difference Chi2-*p*/*t*-test-*p*
Sick leave in last two weeks	No *n* (%)	196 (78)	91 (76)	105 (79)	0.55
	Yes *n* (%)	57 (23)	29 (24)	28 (21)	
Sick leave in last two weeks due to AD	No *n* (%)	222 (88)	104 (87)	118 (89)	na
	Yes *n* (%)	29 (12)	15 (13)	14 (11)	
Mean (sd) number of sick leave days in last two weeks due to AD	0.3 (0.9) (*n* = 251)	0.3 (0.9) (*n* = 119)	0.2 (0.8) (*n* = 132)	0.47	
Sick leave in last year	No *n* (%)	90 (36)	44 (37)	46 (35)	0.73
	Yes *n* (%)	163 (64)	76 (63)	87 (65)	
Sick leave in last year due to AD	No *n* (%)	147 (58) (*n* = 252)	67 (56)	80 (61)	na
	Yes *n* (%)	105 (42)	53 (44)	52 (39)	
Mean (sd) number of sick leave days in last year due to AD	5.7(12.2) (*n* = 252)	7.1 (15.0)	4.4 (8.6) (*n* = 132)	0.09	

na: Not assessed.

**Table 4:** Univariate analyses with factors influencing sick leave in the last two weeks.

Influencing factor	Sick Leave Days in Last Two Weeks (*n* = 251)		
Parameter	Wald ChiSquare Sig.	Odds Ratio	95% Wald Confidence Interval for Odds Ratio Lower-Upper
**Socio demographics**			
Age	0.53	0.99	0.96–1.02
Sex	0.22	1.63	0.74–3.56
Disease duration	0.89	1.00	0.98–1.03
Other chronic diseases	0.74	1.14	0.53–2.47
Numbers of days of work and school per week	0.92	0.98	0.72–1.34
Medium educational level	0.89	0.91	0.24–3.47
High educational level	0.99	0.99	0.26–3.72
**Disease Severity and Quality of Life**			
Disease severity	0.08	1.05	1.00–1.11
Quality of life (total score)	0.00	1.05	1.02–1.07
Quality of life—subscale symptoms	0.02	1.03	1.00–1.05
Quality of life—subscale emotions	0.00	1.03	1.01–1.05
Quality of life—subscale functioning	0.00	1.04	1.02–1.07
**Socio Occupational Factors**			
Symptom interference	0.00	1.22	1.14–1.32
Distress	0.00	1.10	1.04–1.15
Work pressure	0.92	1.00	0.93–1.09
Job dissatisfaction	0.00	1.08	1.02–1.13
Little flexibility	0.61	1.02	0.94–1.11
Stressful home situation	0.00	1.14	1.05–1.24
Avoidance/uncertainty	0.00	1.09	1.03–1.15
Perfectionism/diligence	0.07	0.95	0.90–1.00

**Table 5:** Factors influencing sick leave in the last two weeks based on logistic regression.

Parameter (*n* = 251)	Wald ChiSquare Sig.	Odds Ratio	95% Wald Confidence Interval for Odds Ratio Lower-Upper
(Intercept)	0.00	0.01	
**Disease Severity and Quality of Life**			
Disease severity	0.08	0.93	0.85–1.01
Quality of life—total score	0.51	1.02	0.97–1.06
**Socio Occupational Factors**			
Symptom interference	0.00	1.26	1.13–1.40
Distress	0.91	1.00	0.91–1.09
Job dissatisfaction	0.63	1.02	0.95–1.08
Stressful home situation	0.14	1.08	0.98–1.19
Avoidance/uncertainty	0.71	1.02	0.93–1.11
Perfectionism/diligence	0.00	0.90	0.83–0.96

## 4. Discussion

In this study of adult patients with moderate to severe AD, we showed that 12% of them took sick leave due to AD in the previous two weeks (mean number of days 0.3, sd 0.9) and 42% (mean number of days 5.7, sd 12.2) in the past year. Of all patients, 44% had severely impaired quality of life. Also, 25%–42% showed above mean or high scores on socio-occupational factors. Results indicated that the number of days of sick leave was influenced by a higher level of symptom interference (consisting of influence of severity of complaints on work performance, need of rest, and perceived threshold for return to work), while higher scores on perfectionism/diligence could decrease the number of days of sick leave.

In the Netherlands, about 50% of all employees took sick leave per year [14], with a mean of 7.5 days a year, which is lower than the 64% of this population of AD patients reporting sick leave in the last year. Findings about the number of sick leave days in AD patients differ between published studies. AD patients in this study averaged 5.7 days/year of sick leave compared with lower number of sick leave days in an international [4] and a Canadian study [7]. Two other studies in Denmark and Spain showed a higher number of sick leave days in AD patients [3,5]. Differences in results appear to be attributed to variations in methodology. Data collection included self-reporting questionnaires from our study, the international study with eight countries, the Spanish and Canadian studies [4,5,7] as well as from public resources as in the Danish study [3]. Recall bias may have influenced the precision of the self-reported data about the number of lost work days, especially for information over a longer time period [15]. In addition, the severity of AD of the study population was measured using different tools such as a subjective grading [3,5,7] or physician’s grading [4], which limits comparison between studies.

Based on the results of the univariate analysis, sick leave was influenced both by severity of AD and symptom interference. Multivariate analyses demonstrated that higher symptom interference increases sick leave, and that severity of AD does not. This may be explained by the significant correlation (Pearson’s *r*: 0.56) between both variables. Multivariate analyses without severity of AD decreases the explained variance of the model with only 1%, but in multivariate analyses without symptom interference, the explained variance decreased from 19 to 12% (Cox and Snell R square) and from 37 to 23% (Nagelkerke R square). Therefore, we conclude that symptom interference, which is determined by the influence of disease complaints on work performance, is a better predictor for sick leave than disease severity. This is consistent with the findings of a study in asthma patients which reported that perception of health complaints during social activities increases sick leave [13]. It has been shown that subjective health complaints also increase sick leave in a general working population [16,17].

We observed a novel correlation between perfectionism/diligence and sick leave: perfectionism/diligence could slightly decrease sick leave. Although this particular relationship has not been reported in literature concerning chronic somatic diseases, other factors such as disease control and quality of life were related to sick leave in patients with asthma, psoriasis or COPD [8,9,10,12]. Further research is needed to explain the relationship between sick leave and factors such as perfectionism and symptom interference in patients with AD.

The study population consisted of patients visiting an outpatient department of a university hospital and members or visitors to the website of a patient organization. No significant differences were observed between the two groups regarding disease severity, quality of life and socio-occupational complaints, except for daily functioning (such as sleep, social life and work). AD patients from the patients’ organization scored higher scores in the daily functioning scale, meaning a higher negative impact. The severity (POEM) scores indicate that our study population was representative of patients with moderate to severe AD [18]. However, the diagnosis of AD in patients recruited by the website was reported by the patients themselves and not confirmed by a medical doctor for this study. Besides, response rates were low; 63% (126/200) of the approached patients of the outpatient department gave informed consent while only 25% (172/700) of the people from the patients’ organization did so. Reasons for non-response were often unknown, especially in the group of the patients’ organization. All members of the patients’ organization with a known email address were approached for participation, but about 25% of them were parents of children with AD and they did not fulfil the inclusion criterion concerning adult patients with AD. It is also possible that approached members did not work or attend school and therefore also did not fulfil the inclusion criteria. However, the data suggest that patients who experience greater impact from AD in daily life responded more often because 47% of participants from the patient organization had high scores on the functioning scale of quality of life. Therefore selection bias cannot be ignored, leading to a study population with a higher burden of AD.

The study had a cross sectional design and data were collected for a previous time period, which could have led to recall bias and less reliable results. Moreover, the study may be insufficient to determine influencing factors, since only a minority of patients had sick leave days in the last two weeks. Therefore a prospective design with a larger sample is recommended for further research.

We conclude that sick leave is a common problem in patients with AD. No clear relationship was found between sick leave and disease severity, but symptom interference, the influence of AD complaints on work performance and perfectionism/diligence seemed to influence the amount of sick leave. Since sick leave and loss of productivity during work both contribute to indirect costs of a disease [16], this study highlights the importance of investigating novel approaches to dealing with symptoms at work or school.

## 5. Materials and Methods

### 5.1. Design

A cross-sectional study design was carried out between January and March 2012.

### 5.2. Study Population:

The target population of this study was adult patients with AD. Patients were included if they were diagnosed with AD, were aged 18 years or older, worked or attended school, and had the ability to read or speak Dutch. Patients were recruited at the Dermatology outpatient department of the University Medical Centre Utrecht, The Netherlands, as well as through the website of the Dutch association for patients with AD (VMCE).

### 5.3. Outcome Variables and Instruments

SICK LEAVE: Patients were asked if they took sick leave either in the previous two weeks or year. In addition, they were specifically asked for the number of days of sick leave from work and/or school taken in the previous two weeks and year.

SOCIO-DEMOGRAPHIC FACTORS: These factors were measured by self-reporting about gender, age, educational level, disease duration, other chronic diseases and career.

SOCIO-OCCUPATIONAL FACTORS: The Dutch Work Reintegration Questionnaire [19] measured these factors, which included subscales for the variables distress (sleep behaviour, tiredness, depressive symptoms, distress complaints, appetite); symptom interference (influence of severity of complaints on work performance, need of rest, perceived threshold for return to work); work pressure (job demands, emotional burden, balance home-work); little flexibility (scheduling work tasks, breaks, work pace); job dissatisfaction (work experience, job content, working conditions, social support of manager and colleagues,); avoidance/uncertainty (avoidance behaviour, uncertainty); perfectionism/diligence (handling limits, setting high demands, diligence) and stressful home situation (stressful life events, daily stress). The questionnaire consisted of 78 claims about a patient’s current situation with a four-point Likert-scale (not, sometimes, often, usually). Total score were calculated, and a higher score meant the patient experienced problems more frequently within the concerning variable [20]. For the variable “little flexibility” a higher score should be interpreted as less flexibility. The questionnaire has a high internal consistency and a good test-retest reliability [20,21].

QUALITY OF LIFE: These factors were measured by the Skindex-29 (SD-29) [22,23], which consists of the subscales symptoms, emotions and daily functioning. Patients were asked 30 questions about the previous week on a five-point Likert-scale (never, rarely, sometimes, often, always). Higher scores signify a more severe negative impact on the quality of life [24]. The Skindex-29 has moderate to good psychometric properties [25,26].

DISEASE SEVERITY: Disease severity was measured by the Patient-Oriented Eczema Measure (POEM) [27,28]. The questionnaire consisted of seven questions about the previous week with a five-point Likert-scale answer possibility (0 days, 1–2 days, 3–4 days, 5–6 days, all days of the week). A higher total score indicates a higher disease severity, with a maximum total score of 28. The questionnaire has moderate to good psychometric properties [27] and moderate sensitivity to change [28].

Data were collected via a questionnaire to be completed either on the computer or on paper.

### 5.4. Data Analysis

DESCRIPTIVES: Number (percentage) of patients who took sick leave in the previous two weeks or last year, as well as the number of days of sick leave due to AD (mean, sd) were described. In cases in which the amount of lost days in the previous two weeks was higher than that of the previous year, the amount of lost days in the previous year was calculated by the sum of the amount of lost days in the previous two weeks and that of the previous year. Socio-demographic characteristics are presented as descriptives or frequencies. The scores of the eight subscales of the Work Reintegration Questionnaire were dichotomized and depicted as percentages of patients with low to mean scores and above mean scores, based on a table with standard scores of Dutch employees who took sick leave [19]. Above mean scores for the subscales are: distress ≥29; symptom interference ≥31; work pressure ≥16; little flexibility ≥17; job dissatisfaction ≥26; avoidance/uncertainty ≥24; perfectionism/diligence ≥37 and stressful home situation ≥15. Scores on the Skindex-29 were also dichotomized, based on cut-off scores for severely impaired health related quality of life [23]. The cut-off scores are for the subscale symptoms ≥52, emotions ≥39, functioning ≥37, and for the overall score ≥44 points. The total scores of POEM were presented as mean (sd) total score and range. Severity range was based on proposed banding [18] in clear/almost clear (score 0–2); mild (score 3–7); moderate (score 8–16); severe (score 17–24); very severe (score 25–28).

LOGISTIC REGRESSION: Logistic regression was used to analyse predictors of sick leave at work or school in the last two weeks in patients with AD. Factors used for modelling the regression equation were selected by univariate logistic regression. Factors with a significance level of *p* ≤ 0.20 were used in the multivariate logistic regression. All analyses were conducted with IBM SPSS statistics 20. Only completed questionnaires were included in the analyses.

SAMPLE SIZE: A sample size of 270 patients provided reliable evidence for the primary research question based on a 95% confidence level; a response distribution of 50%; an estimated eligible, population size of 900; and a 5% margin of error [29].

### 5.5. Ethical Approval

All patients were carefully informed about the study and gave their informed consent. The study was not covered by the Medical Research Involving Human Subjects Act which was confirmed by the Medical Research Ethics Committee of the UMC Utrecht, the Netherlands (protocol number 11-556/C).

## 6. Conclusions

Sick leave in patients with AD is a common problem and symptom interference and perfectionism/diligence appeared to influence it. Novel approaches are needed to deal with symptoms at work or school to reduce the amount of sick leave due to AD.
